# A Major Downregulation of Circulating microRNAs in Zika Acutely Infected Patients: Potential Implications in Innate and Adaptive Immune Response Signaling Pathways

**DOI:** 10.3389/fgene.2022.857728

**Published:** 2022-06-01

**Authors:** Ana Carolina Carvalho-Silva, Almir Ribeiro Da Silva Junior, Vagner Oliveira-Carvalho Rigaud, Waleska Kerllen Martins, Verônica Coelho, Irmtraut Araci Hoffmann Pfrimer, Jorge Kalil, Simone Gonçalves Fonseca, Edecio Cunha-Neto, Ludmila Rodrigues Pinto Ferreira

**Affiliations:** ^1^ RNA Systems Biology Laboratory (RSBL), Departmento de Morfologia, Instituto de Ciências Biológicas, Universidade Federal de Minas Gerais, Belo Horizonte, Brazil; ^2^ Programa de Pós-Graduação em Biologia Celular, Universidade Federal de Minas Gerais (UFMG), Belo Horizonte, Brazil; ^3^ Laboratory of Immunology, Heart Institute (InCor) School of Medicine, University of São Paulo, São Paulo, Brazil; ^4^ Institute for Investigation in Immunology, iii-INCT (National Institute of Science and Technology), São Paulo, Brazil; ^5^ Division of Clinical Immunology and Allergy, School of Medicine, University of São Paulo, São Paulo, Brazil; ^6^ Instituto de Química, Universidade de São Paulo, São Paulo, Brazil; ^7^ Universidade Anhanguera, São Paulo, Brazil; ^8^ Pontifícia Universidade Católica de Goiás, Goiânia, Brazil; ^9^ Instituto de Patologia Tropical e Saúde Pública, Universidade Federal de Goiás, Goiânia, Brazil; ^10^ National Institute of Science and Technology for Vaccines (INCTV), Belo Horizonte, Brazil; ^11^ Centro de Tecnologia de Vacinas, Universidade Federal de Minas Gerais, Belo Horizonte, Brazil

**Keywords:** Zika infection, systems biology, microRNA profiling, target prediction, viruses

## Abstract

Zika virus (ZIKV) is an arbovirus mainly transmitted by mosquitos of the genus *Aedes*. The first cases of ZIKV infection in South America occurred in Brazil in 2015. The infection in humans causes diverse symptoms from asymptomatic to a syndrome-like dengue infection with fever, arthralgia, and myalgia. Furthermore, ZIKV infection during pregnancy is associated with fetal microcephaly and neurological disorders. The identification of host molecular mechanisms responsible for the modulation of different signaling pathways in response to ZIKV is the first step to finding potential biomarkers and therapeutic targets and understanding disease outcomes. In the last decade, it has been shown that microRNAs (miRNAs) are important post-transcriptional regulators involved in virtually all cellular processes. miRNAs present in body fluids can not only serve as key biomarkers for diagnostics and prognosis of human disorders but also contribute to cellular signaling offering new insights into pathological mechanisms. Here, we describe for the first time ZIKV-induced changes in miRNA plasma levels in patients during the acute and recovery phases of infection. We observed that during ZIKV acute infection, among the dysregulated miRNAs (DMs), the majority is with decreased levels when compared to convalescent and control patients. We used systems biology tools to build and highlight biological interactions between miRNAs and their multiple direct and indirect target molecules. Among the 24 DMs identified in ZIKV + patients, miR-146, miR-125a-5p, miR-30-5p, and miR-142-3p were related to signaling pathways modulated during infection and immune response. The results presented here are an effort to open new vistas for the key roles of miRNAs during ZIKV infection.

## Introduction

The Zika virus (ZIKV) is an arbovirus member of the Flaviviridae family that can be carried and spread by mosquitoes belonging to the genus *Aedes*. The first Zika epidemic in South America occurred in 2015 in the northeastern states of Brazil (Campos et al., 2015; Hamel et al., 2015; Zanluca et al., 2015). About one in five people infected with the Zika virus become symptomatic with characteristic clinical symptoms such as acute onset fever with rash, arthralgia, and conjunctivitis. Other commonly reported symptoms include myalgia, headache, retroorbital pain, and vomiting (Oehler et al., 2014; Musso et al., 2015). Previous studies have shown that ZIKV has a neuronal and glia tropism and can be also associated with neurological disorders (for example, Guillain–Barré syndrome and myelitis) (Oehler et al., 2014). Identification of viral RNA in amniotic fluid and maternal milk demonstrated that transmission may also occur from mother to fetus during pregnancy causing dysfunction and microcephaly of the developing fetal brain (De Carvalho et al., 2016). Knowledge about the pathogenesis of ZIKV infection is still limited, and an urgent scientific effort is needed to understand, develop, and identify factors associated with clinical outcomes. microRNAs (miRNAs or miRs) are single-stranded non-coding RNA molecules of ∼22 nucleotides in length capable of controlling gene expression at the transcriptional and translational levels by repressing target mRNAs (Alvarezbuylla et al., 2007; van Rooij, 2011). miRNAs have been detected stably circulating in various body fluids, such as saliva, urine, serum, and plasma of control or diseased individuals being extensively reported as non-invasive diagnostic and prognostic biomarkers (Mitchell et al., 2008; Ouyang et al., 2016). Here, we describe for the first time the circulating miRNA profiling in plasma samples from ZIKV-infected individuals during acute (ZIKV+) and the recovery phase (RECZIKV+) compared to control donors (CONTROL). We have found that ZIKV + patients had a significant number of had a significant number of dysregulated miRNAs (DMs) in plasma, with most of them with decreased levels than RECZIKV+ and CONTROL individuals. To better understand and predict the potential impact of this miRNA dysregulation during Zika infection we used computational analysis to identify the potential targets of the DMs (DM Targets). Further pathway enrichment and functional analyses were performed to identify potential upstream regulators of DMs during Zika infection. Finally, DM-target networks were built, and central node molecules were found at ZIKV+ and RECZIKV + networks around the main enriched canonical pathways observed for each group. We present here a holistic view and highlight the potential role of circulating miRNAs during the response to acute infection and the disease recovery phase of ZIKV infection.

## Materials and Methods

### Ethics Statement

This study was approved by the Institutional Review Board from Pontifical Catholic University of Goiás (CEP—Research Ethics Committee), under protocol number 46073815.9.0000.00370. All subjects were invited to participate in the study after being explained about the research and were at least 18 years old. All study subjects signed a written informed consent form before the interview and blood collection in accordance with the Declaration of Helsinki. The patients with Zika fever-like symptoms were also interviewed in a private room and answered a written questionnaire informing them about the day of symptom onset, types of symptoms, and demographic information.

### Study Groups and Sample Collection

Our study included a total of 52 individuals aged 18–68 (mean 36 years) from Goiânia city, state of Goiás (GO), Brazil. All individuals tested negative for dengue and chikungunya. Of these 52 participants (Supplementary Table S1) 25 were potential blood donors recruited from the Center of Serology and Immunohematology of Goiânia-GO–Brazil, aged 27–53 (mean 36.4 years) and displaying negative blood tests for several infectious diseases (CONTROL). The remaining 27 participants showed Zika fever-like acute symptoms during the outbreak of ZIKV infection in Brazil between January and May 2016. All 27 patients were positive for ZIKV at real-time RT-PCR test (ZIKV+). A first plasma collection of ZIKV-infected subjects was done at enrollment, 2–9 days after symptom onset. A second collection was done from 6 ZIKV-infected subjects 2–3 weeks after the first sample, for evaluating the recovery phase (RECZIKV+) (Table 1). From these, 18 samples, we randomly selected a total for miRNA profiling: 6 control individuals (CONTROL), 7 ZIKV+ and 5 RECZIKV+. Plasma was isolated from blood samples collected into EDTA-coated Vacutainer tubes (Becton & Dickinson, United States), centrifuged at 1,200x g for 10 min, and stored at −80°C freezer until the analysis.

**TABLE 1:** Characteristics of individuals/patients from the miRNA profiling groups.

Patient code	Group	Gender	Age (years)	Days (symptom onset to sampling)	qPCR ZIKV (C.T.)**
C1	CONTROL	F	34	None	N.A
C2	CONTROL	F	36	None	N.A
C3	CONTROL	F	39	None	N.A
C4	CONTROL	F	36	None	N.A
C5	CONTROL	F	53	None	N.A
C6	CONTROL	F	37	None	N.A
ZIKV+1	ZIKV+	F	36	4	38.92
ZIKV+2	ZIKV+	F	34	3	31.25
ZIKV+3	ZIKV+	F	49	4	35.82
ZIKV+4	ZIKV+	F	36	2	35.85
ZIKV+5	ZIKV+	M	33	9	29.28
ZIKV+6	ZIKV+	M	28	2	27.21
ZIKV+7	ZIKV+	M	24	3	37.88
RECZIKV+1	ZIKV+	F	39	>15	N.A
RECZIKV+2	ZIKV+	F	36	>15	N.A
RECZIKV+3	ZIKV+	F	36	>15	N.A
RECZIKV+4	ZIKV+	F	34	>15	N.A
RECZIKV+5	ZIKV+	F	49	>15	N.A

Gender: F = female M = male; C.T, cycle threshold; none = no symptoms; N.A, not amplified. Samples used for miRNA, profiling: six control individuals (CONTROL); seven ZIKV+ and five RECZIKV + patient samples.

### Detection of ZIKV Infection by Real-Time RT-PCR

Viral RNA detection and quantification were performed by extracting RNA from whole blood using the QIAamp Viral RNA Mini Kit (Qiagen, Hilden, Germany), according to the manufacturer’s instructions. Real-time RT-PCR for Zika virus was performed using a kit (Bioclin®, Bio gene Zika virus PCR- K-203–6). The following primers and probes were used: ZIKV-F, 5’-CCG​CTG​CCC​AAC​ACA​AG-3’; ZIKV-R, 5’-CCA​CTA​ACG​TTC​TTT​TGC​AGA​CAT-3’; ZIKV-P, 5’FAM-AGCCTACCTTGACAAGCAGTCAGACACTCAA-BHQ1-3, developed according to Lanciotti et al. (2007) with modifications (Lanciotti et al., 2008). The RT-PCR was performed following the manufacturer’s instructions as published before. Also, real-time RT-PCR for chikungunya and dengue was performed using probe sequences described before (Barros et al., 2018).

### Assessment of Hemolysis

Before miRNA profiling, we checked plasma for hemolysis by measuring absorbance at 350–650 nm by spectrophotometry (Nanodrop 2000 spectrophotometer, Thermo Scientific, Waltham, Massachusetts, United States). Samples were classified as hemolyzed if the OD414 exceeded a value of 0.2 (Kirschner et al., 2011). Previously hemolyzed samples were not used in the study.

### Purification of Plasma RNA Enriched in miRNAs

Total RNA was purified from plasma using the Qiagen miRNeasy^®^ Mini Kit. An aliquot of 200 μl of plasma per sample was thawed on ice and centrifuged at 3,000 x g for 5 min at 4°C. The aliquot was transferred to a new tube and 750 μl of a Qiazol mixture containing 3 μl of a spike-in, a synthetic miRNA from *Caenorhabditis elegans* (cel-miR-39) at 1.6 × 108 copies/μl was added to each plasma sample (used as normalization in individual assay analyses). The tube was mixed and incubated for 5 min followed by the addition of 200 μl chloroform. The tube was mixed, incubated for 2 min, and centrifuged at 12,000 x g for 15 min at 4°C. The upper aqueous phase was transferred to a new microcentrifuge tube and 1.5 volume of 100% ethanol was added. The contents were mixed thoroughly and 750 μl of the sample was transferred to a Qiagen RNeasy^®^ Mini spin column in a collection tube followed by centrifugation at 15,000 x g for 30 s at room temperature. The process was repeated until all remaining samples had been loaded. The Qiagen RNeasy^®^ Mini spin column was rinsed with 700 μl of Qiagen RWT buffer and centrifuged at 15,000 x g for 1 min at room temperature followed by another rinse with 500 μl of Qiagen RPE buffer and centrifuged at 15,000 x g for 1 min at room temperature. A rinsing step (500 μl of Qiagen RPE buffer) was repeated 2X. The Qiagen RNeasy^®^ Mini spin column was transferred to a new collection tube and centrifuged at 15,000 x g for 2 min at room temperature. The Qiagen RNeasy^®^ Mini spin column was transferred to a new microcentrifuge tube and the lid was left uncapped for 1 min to allow the column to dry. Total RNA was eluted by adding 14 μl of RNase-free water to the membrane of the Qiagen RNeasy^®^ mini spin column and incubating for 1 min before centrifugation at 15,000 x g for 1 min at room temperature. The concentration of the final material was determined by measuring the A260/A280 ratio using a NanoDrop ND-2000 apparatus (Thermo Scientific, Waltham, Massachusetts, United States). The RNA was stored in a −80°C freezer.

### miRNA Profiling

miRNA profiling of 377 miRNAs was carried out following the manufacturer’s protocol (Thermofisher). Briefly, a multiplexed RT reaction was performed followed by a pre-amplification using 2.5 μl of the cDNA and 22.5 μl of the pre-amplification master mix with cycling conditions that included 10 min at 95°C, 2 min at 55°C, and 2 min at 72°C, 12 cycles of 15 s at 95°C and 4 min at 60°C, and 10 min at 99.9°C. Quantitative real-time RT-PCR was done utilizing pre-printed TLDA microfluidic cards (Human Card A v3, format 384). The sample/master containing the Megaplex pool was loaded into the cards, centrifuged, and mechanically sealed with the Applied Biosystems sealer device. The real-time PCR reaction was carried out on a QuantStudio™ 12K Flex (Applied Biosystems) real-time machine, using the cycling conditions recommended by the manufacturer.

### miRNA Statistical and Unsupervised Analyses

To analyze the miRNA levels we uploaded the real-time generated raw data files (file extension. EDS) in a Thermofisher Cloud software v1.0 (Connect, https://www.thermofisher.com/br/en/home/cloud.html). This software exploits an independent samples *t*-test to compare Ct data to one randomly selected representative reference control sample using a two-tailed *p*-value value of 0.05 and relative miRNA levels are presented as fold change. The data files were first pre-processed by using automatic baseline corrections and manually checked for each assay if the threshold cycle (Ct) value corresponded to the midpoint of the logarithmic amplification curve. miRNAs with a mean Ct > 38 and detected in <80% of all samples were considered below the detection level and excluded from further analysis. The comparative threshold cycle method was used to calculate the relative miRNA levels after global mean normalization (ΔCt) (Mestdagh et al., 2009). The sample unsupervised analysis was performed by hierarchical clustering using Manhattan distance and average linkage for column, and correlation distance and average linkage for row, and represented as a heatmap with ΔCt values for 113 miRNAs (rows), and 18 columns (samples). The PCA of miRNA levels was performed for all samples and the same set of miRNAs was used in the hierarchical clustering. PCA was performed by using a median centering of the data set. The *x*-axis corresponds to principal component 1 (PC1) and the *y*-axis to principal component 2 (PC2) and the percentages of variance in both. Both hierarchical clustering and PCA were built using the ClustVis web tool.

### Quantitative Real-Time PCR Analysis of miR-142-3p Plasma Levels

After total RNA isolation, using the miRNeasy Serum/Plasma kit (Qiagen), cDNA was synthesized by reverse transcription using a fixed volume of RNA (2 μl) and the TaqMan microRNA Reverse Transcription kit (Life Technologies), according to the manufacturer’s instructions. The circulating levels of miR-142-3p and a synthetic RNA (cel-miR-39) (added during RNA purification) were measured by RT-qPCR using 1.33 μl of the cDNA and miRNA-specific stem-loop primers provided by TaqMan microRNA Assays kit (Life Technologies). Quantitative PCR reactions were performed in triplicate on a QuantStudio 12K Flex (Life Technologies), according to the following program: 10 min at 95°C, 40 cycles of 15 s at 95°C and 60 s at 60°C. Values were normalized to the cel-miR-39 spike and analyzed by the comparative method of Ct (2-ΔΔCt). A threshold cycle (Ct) was observed in the exponential phase of amplification, and quantification of relative levels was performed using standard curves for miR-142-3p and cel-miR-39. Reactions were performed in triplicate and Ct values were averaged for the replicates. The levels were calculated as the mean ± s.d. for each group as individual data points and the following formula was used: relative expression (fold change over CONTROL group samples) = 2^−(∆CtA–∆CtB)^, where Ct is the cycle threshold as previously described (Livak and Schmittgen, 2001). The same was calculated for the CONTROL group, subtracting its mean by each individual data point, so we could plot small variations close to 1 of fold change. Groups were compared by a non-parametrical Kruskal Wallis test. The miRNAs were considered dysregulated if *p* < 0.05 and absolute fold change (FC)≥1.5.

### Target Prediction, Canonical Pathway Enrichment, Function, and Network Analysis

The software Ingenuity Pathways Analysis (IPA) (Qiagen, United States) was used in all computational analyses. The target prediction was performed using an IPA tool called “target filter” which relies on four different database algorithms: TargetScan, TarBase, miRecords, and Ingenuity Expert Findings. In our target prediction analysis, we only considered the DM targets that were experimentally validated and highly predicted as targets, based on the content of date 2019–12. The canonical pathway enrichment, function, and network analysis were performed by uploading these target lists identified for each group on IPA software. The significance of the association between each list and the canonical pathway and the relationship between two node molecules in the built networks was measured by Fisher’s exact test. As a result, Benjamini–Hochberg method adjusted *p*-values (<0.05) were obtained, determining the probability that the association between the targets in our data set and the canonical pathways identified and networks generated can be explained by chance alone.

### Bioinformatics and Statistical Analysis

MicroRNA profiling statistical analysis was carried out by using the Thermo Fisher Cloud software (Connect) which exploits an independent samples t‐test to compare ΔΔCt data to one randomly selected representative reference control sample using a two‐tailed *p*‐value threshold of 0.05 with adjustment for false discovery rate with the Benjamini–Hochberg method. The comparative threshold cycle method was used to calculate the relative miRNA levels after global normalization. The statistical significance threshold was defined as *p* < 0.05 and FC ≥ 1.5. Unsupervised hierarchical clustering was performed using squared Euclidean as distance measure and Ward’s method for linkage analysis and Z score normalization. The PCA plot was performed using all probe sets, by using a median centering of the data set.

## Results

### Unsupervised Analyses and Identification of DMs in Plasma of Patients Infected With ZIKV

**FIGURE 1:**
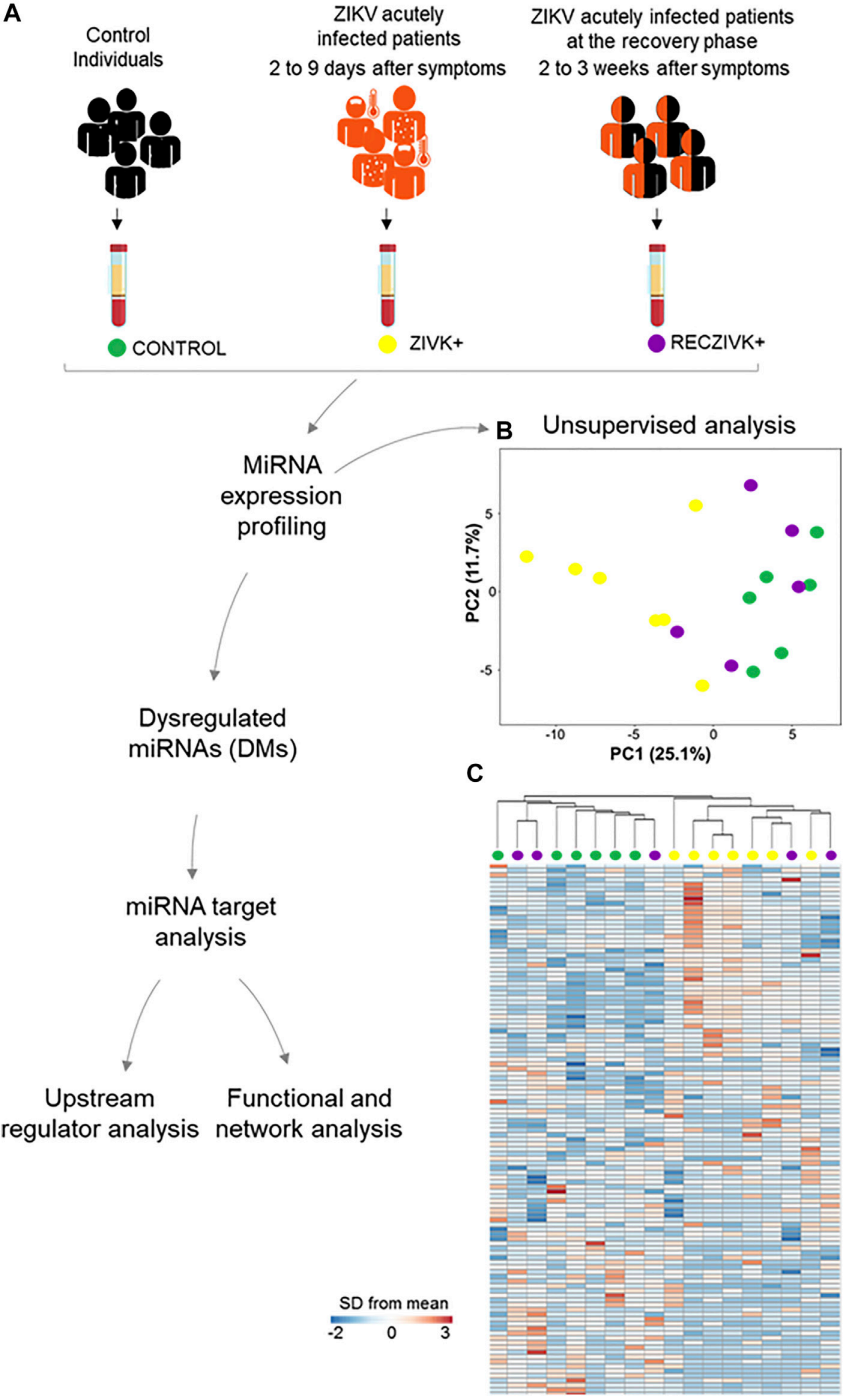
**(A–C)** Workflow and unsupervised analysis of miRNAs. **(A)** Sample collection, distribution of the studied groups, data processing, and analysis of miRNA profiles in Zika acutely infected patients (ZIKV+; yellow dots) and at the recovery phase of infection (RECZIKV+; magenta dots) compared to control individuals (CONTROL; green dots). **(B)** Principal component analysis (PCA) of miRNA based on all samples and 113 miRNAs by using a median centering of the data set. **(C)** Heatmap and hierarchical clustering were performed with all samples using Manhattan distance and average linkage for columns, and correlation distance and average linkage for rows, and represented as a heatmap with ΔCt values for 113 miRNAs (rows) and 18 samples (columns). The color scale illustrates the fold change in microRNA levels relative to all groups; red and blue represents increased and decreased levels respectively.

We performed profiling of 377 miRNAs from plasma of Zika-infected patients at acute (ZIKV+) and the recovery phase (RECZIKV+) of the disease compared to control individuals (CONTROL) from the same endemic area. Figure 1A summarizes the experiment workflow showing the steps from blood collection to computational analyses. Briefly, we performed miRNA profiling and identified the list of DMs using cloud-based software, as described in detail in the material and methods section. The miRNA profiles from each comparison were used to find their predicted targets. The lists of predicted targets were used in the functional, upstream regulator, and network analyses. Unsupervised analysis: PCA (Figure 1B) and hierarchical clustering (Figure 1C) were performed based on the detection of 113 miRNAs with a higher level variance (rows) from each one of the 18 samples (columns) (Figure 1C). miRNA profiles from ZIKV+ (yellow dots) and CONTROL (green dots) were segregated in inter-group clusters, while the RECZIKV + group (magenta dots) did not segregate from the other two. One sample clustered within the CONTROL group and the other two within the ZIKV + group. Both PCA and hierarchical clustering indicated that miRNA profiles were specific only to ZIKV+ and CONTROL groups with RECZIV + group sharing miRnome profile similarities with these other two groups.

### Identification of DMs and DM Putative Target Prediction

**FIGURE 2:**
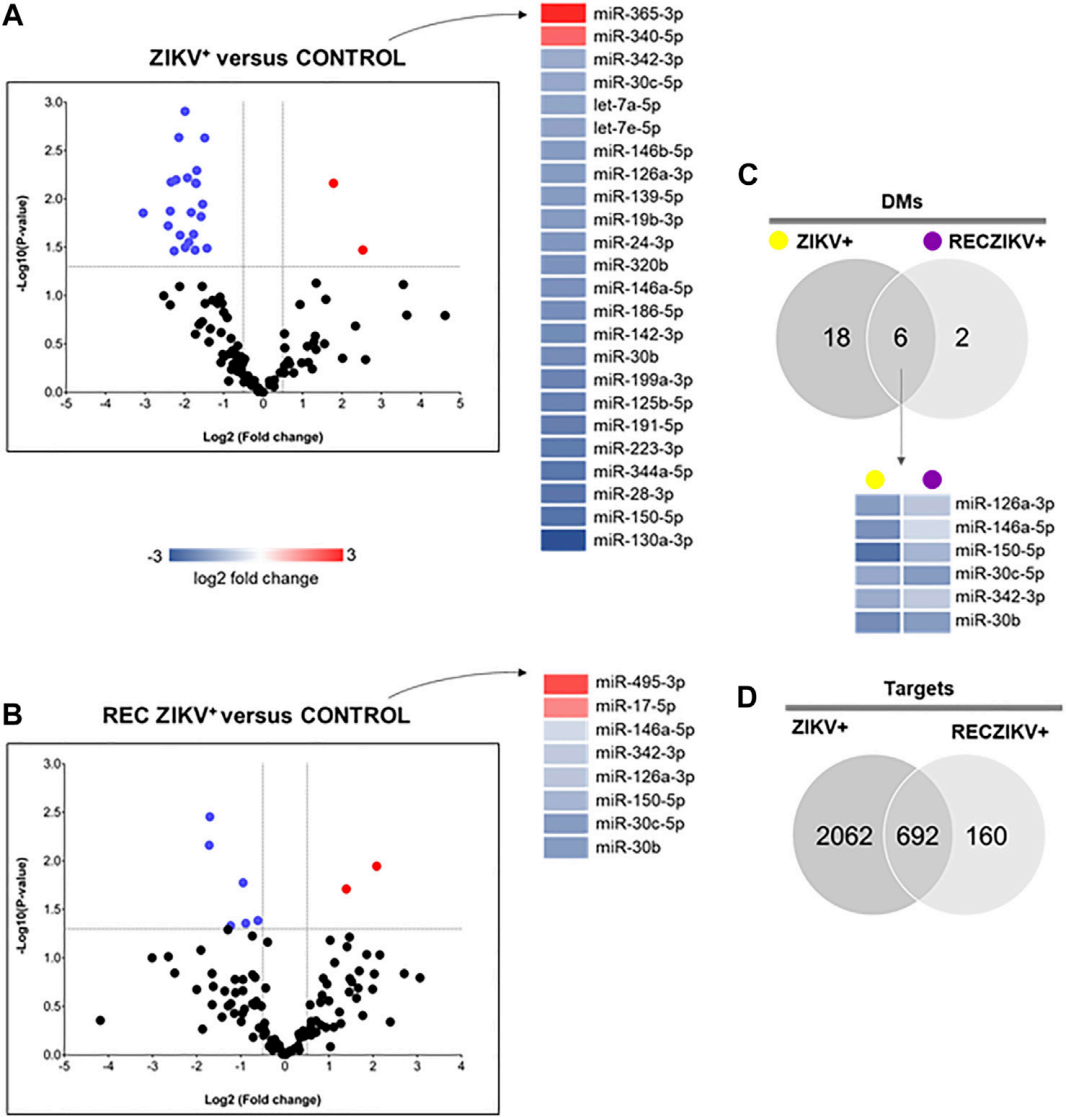
**(A–D)** Profiling of dysregulated miRNA in ZIKV+, RECZIKV+, CONTROL, and their total targets. **(A,B)** Volcano plot of altered microRNAs from the samples of ZIKV + versus CONTROL group and RECZIKV + versus CONTROL group. The color scale illustrates the log2 fold change in microRNA levels relative to all groups; red and blue represents increased and decreased levels, respectively. **(C)** Venn diagram demonstrated the number of DMs from each group and six miRNAs are common between the ZIKV+ and RECZIKV+, all of them are decreased. **(D)** Venn diagram of the putative targets predicted for increased- and decreased miRNAs in ZIKV+ and RECZIKV+.

miRNA profiling revealed the highest number of DMs in the ZIKV + compared to the CONTROL group (Figures 2A–C). The Volcano plot representations show 24 DMs for ZIKV + versus CONTROL, with most miRNAs (22) levels decreased and only 2 increased (Figure 2A). We identified 8 DMs in RECZIKV+ compared to CONTROL groups. From these 8 DMs, 2 were with higher levels and 6 wih lower levels in patients in the recovery phase of ZIKV infection (Figure 2B). The Supplementary Table S2, S3 depict the list of all DMs in each comparison. Figure 2C shows a Venn diagram with the number of DMs from each group, ZIKV+, and RECZIKV + compared to CONTROL, and the 6 DMs shared between the two groups (Supplementary Table S4). All shared miRNAs were decreased in both groups. Next, we used a miRNA target prediction tool from Ingenuity Pathway Analysis software (IPA) to screen the putative targets on each list of DMs. The target prediction analysis finds targets (RNAs) with complementary sequences to the miRNA seed region (nucleotides 2-8 from the 5’ end of the mature sequence) in their 3´UTR. As miRNAs with the same seed sequence usually target the same RNAs, IPA software clusters together with the mature miRNAs that share the same 7-nucleotide seed sequence into one entity or “node” to increase the specificity of targeting information. Three pairs out of the 24 DMs from the ZIKV + group share the same seed sequence: let-7a-5p and let-7e-5p (seed sequence GAGGUAG), miR-146a-5p and miR-146b-5p (seed sequence GAGAACU) and miR-30b and miR-30c-5p (seed sequence GUAAACA). The set of miR-30b and miR-30c-5p is also dysregulated in the RECZIKV + group. The target prediction analysis also considers as targets only those that have been highly predicted as targets (from TargetScan database) and/or experimentally validated as targets (from miRecords, TarBase, and direct acquisition from the literature by Ingenuity knowledge Base -IKB). From the list of 24 DMs identified in ZIKV + patients, we found targeting information for 21 of them. We thus obtained a list of 2,754 targets (Supplementary Table S5) of the 21 DMs in ZIKV+. For the RECZIKV+, from the list of 8 DMs after filtering, we obtained a list of 7 DMs targeting a total number of 852 targets (Supplementary Table S6). A Venn diagram in Figure 2D shows that ZIKV+ and RECZIKV + have 692 targets in common (Supplementary Table S7).

### Target Set Enrichment Analysis Reveals Potential Pathways Regulated by miRNAs During Acute and Recovery Phase of Zika Infection

**FIGURE 3:**
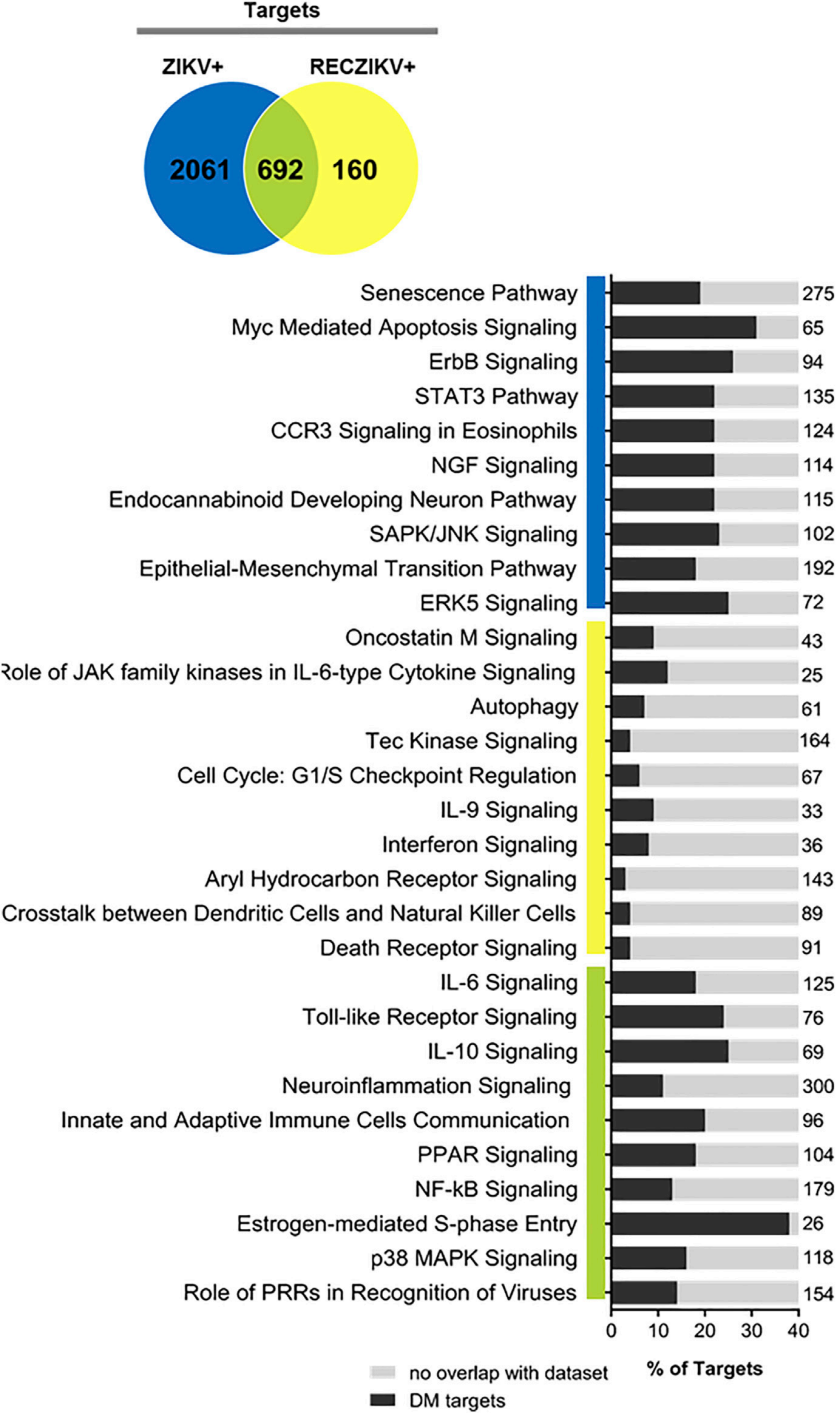
Ingenuity Pathway Analysis (IPA) canonical pathways most significantly enriched in ZIKV+ (blue color), RECZIKV+ (yellow color), and both groups (green color). The stacked bar chart displays the percentage of DM-target molecules present in each pathway. The numerical value to the right of each bar name represents the total number of molecules in that canonical pathway. The Benjamini–Hochberg method was used to adjust the right-tailed Fisher’s exact test *p*-value, which was always (*p* < 0.05).

To predict the canonical pathways enriched and potentially regulated by the DMs from each group we carried out a functional analysis using IPA software. In Figure 3, we show the top 10 most enriched canonical pathways (Benjamini–Hochberg adjusted *p* values <0.05) for a specific list of targets from each group (ZIKV+ in blue, RECZIV+ in yellow, and the shared targets in green). The stacked bar charts show the percentage of targets in each one of the enriched pathways for each group and the number at the right of each bar represents the number of molecules in that given pathway. The analysis of the 692 shared targets between ZIKV+ and RECZIKV+ (in green) showed an overrepresentation of canonical pathways related to immune response such as *pattern recognition of pathogens by the innate immune response*, *toll-like receptor signaling*, and the *role of PRRs in recognition of viruses*. Also, there is an enrichment of pathways related to inflammation, for example, *IL6*, *IL10 signaling,* and most importantly neuroinflammation. Furthermore, we found pathways related to response to cell stress such as *apoptosis and senescence pathways* for the ZIKV + exclusive targets (in blue), and canonical pathways such as *oncostatin M*, *autophagy, and interferon signaling* among others for the RECZIKV+ (yellow).

### DM-Target Networks Revealed Key Molecules During ZIKV + Infection

**FIGURE 4:**
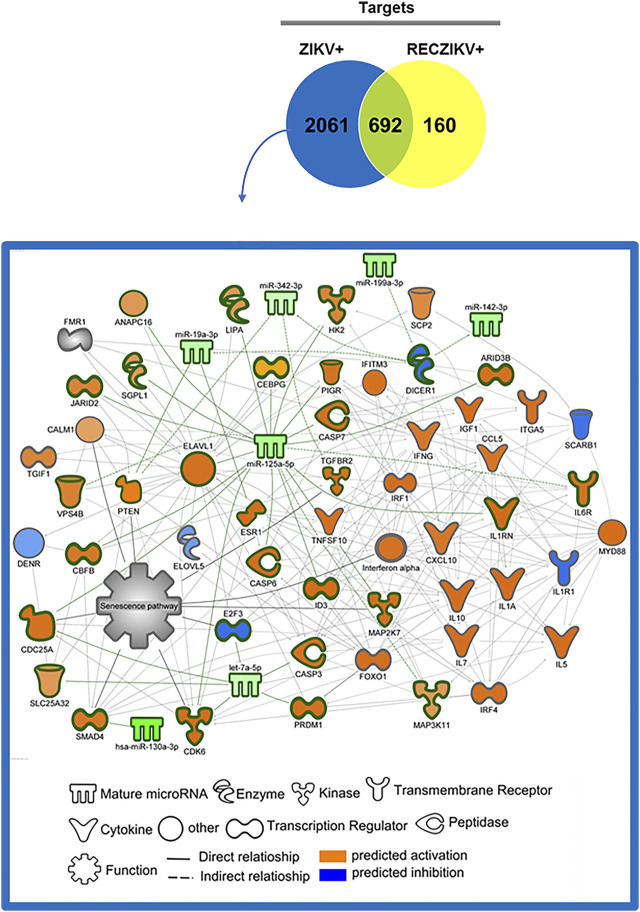
DM and molecule network related to senescence pathway regulation in ZIKV+. The network was built using IPA software. Each molecule was represented as a node, and the biological relationship between two nodes is represented as an edge (line). All edges are supported by at least one reference from the literature or canonical information stored in the Ingenuity Knowledge Base (*IKB*). The central nodes are connected to multiple other molecules. The ZIKV + network showed some molecules as central nodes: interferon-alpha, CXCL10, interferon-gamma (IFNG), interferon regulatory factor 1 (IRF1), miR-125a-5p and its experimentally validated target *ELAV* like RNA-binding protein 1 (ELAV1). The molecules are represented in a gradient of orange or blue based on the prediction status, activated or inhibited, respectively.

**FIGURE 5:**
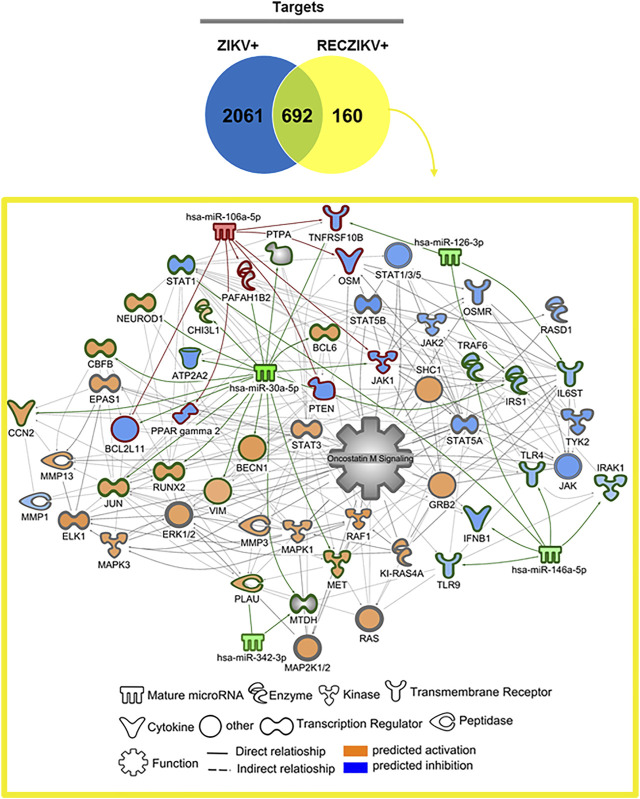
DM and molecule network related to oncostatin M pathway regulation in RECZIKV+. The network was built using IPA software. Each molecule was represented as a node, and the biological relationship between two nodes is represented as an edge (line). All edges are supported by at least one reference from the literature or canonical information stored in the Ingenuity Knowledge Base (IKB). The molecule network is directly or indirectly related to the oncostatin M signaling canonical pathway. MiR-30a-5p is a central node targeting some other node molecules like signal transducer and activator of transcription 1 (STAT1) and 3 (STAT3), activator protein 1 (JUN), and neuronal differentiation 1 (NEUROD1).

To investigate the possible role of miRNAs in regulating key targets during the acute and recovery phases of Zika infection, we built DM-target networks for both groups (ZIKV+ and RECZIKV+). IPA has a graphical database of networks of interacting molecules (Ingenuity Knowledge Base, IKB). Molecules (genes, proteins) are represented as nodes, and biological relationships between nodes are represented as edges (lines). All connections are supported by at least one reference from the literature or canonical information stored in the IKB. The built networks and prediction analysis revealed the potential role/connection of DMs and their targets in regulating the top predicted canonical pathways in each group: *senescence pathway* in the ZIKV + group (Figure 4) and *oncostatin M signaling* in the RECZIKV + group (Figure 5). The molecules are represented in a gradient of red or green based on their fold change (increased or decreased, respectively) and orange or blue (predicted to be activated or inhibited). Each node shape represents one type of molecule. The combined biological interaction in both built networks revealed genes that are central nodes connected to multiple other molecules. The ZIKV + network showed some molecules as central nodes: for example, interferon-alpha, CXCL10, interferon-gamma (IFNG), interferon regulatory factor 1 (IRF1), miR-125a-5p and its experimentally validated target ELAV like RNA-binding protein 1 (ELAV1). For the RECZIKV + network, miR-30a-5p is a central node targeting some other node molecules such as signal transducer and activator of transcription 1 (STAT1) and 3 (STAT3), activator protein 1 (JUN), and neuronal differentiation 1 (NEUROD1), molecules directly or indirectly related to *oncostatin M signaling*.

### The Hematopoietic Cell-Specific miR-142-3p Downregulated During Acute Zika Infection Potentially Regulates Viral Entry Endocytic Pathways

**FIGURE 6:**
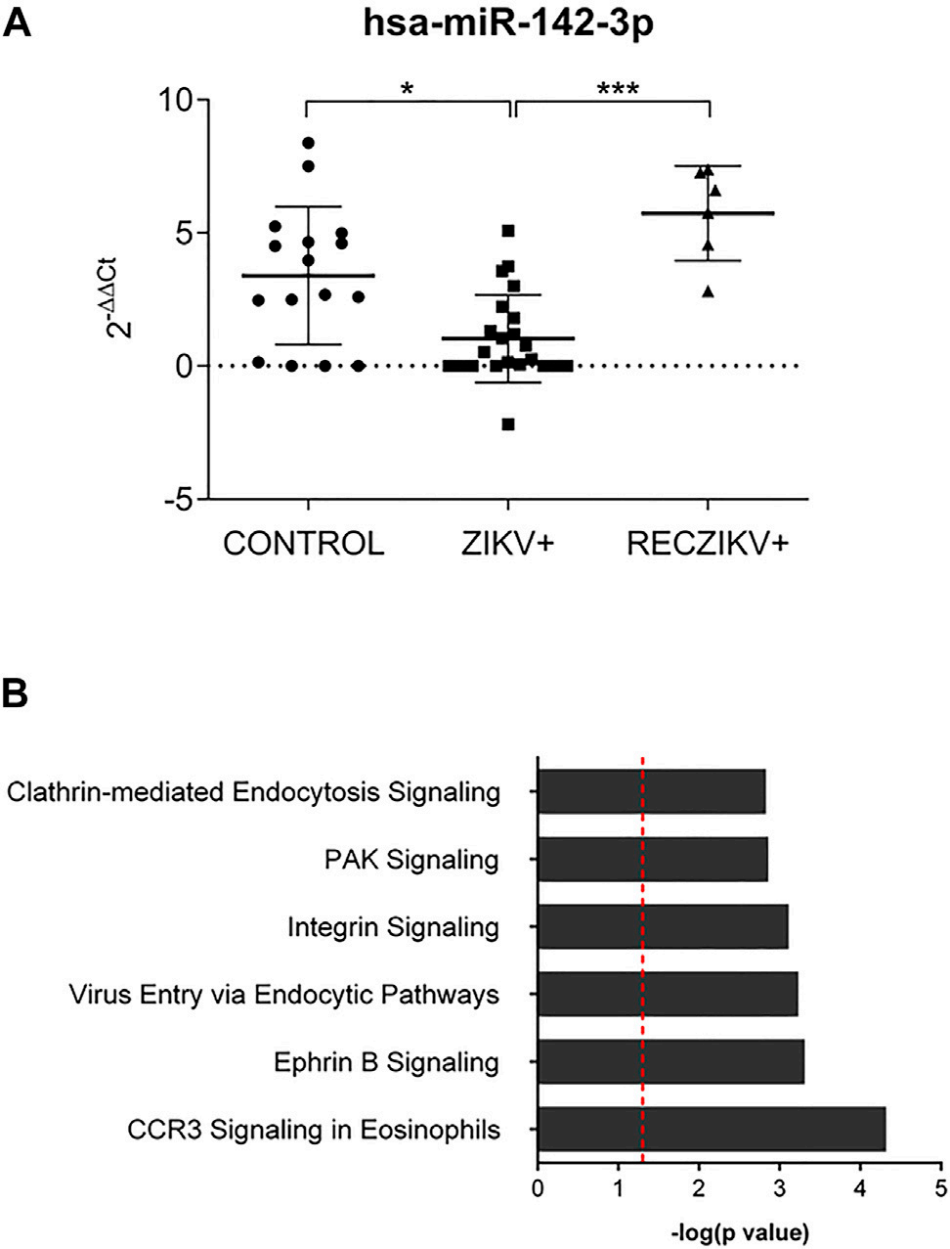
**(A,B)** Evaluation of miR-142-3p in the ZIKV +, RECZIKV + and CONTROL groups. **(A)** The qPCR validation was performed with 22 ZIKV+, 16 CONTROL and 6 previously analyzed RECZIKV + plasma samples. MiR-142-3p is significantly reduced in ZIKV + compared to CONTROL (*p < 0.05) and RECZIKV+ (***p < 0.005). The levels were calculated as the mean ± s.d. for each group as individual data points using relative expression (fold change over CONTROL) by the 2^−ΔΔct^ method followed by a non-parametrical Kruskal–Wallis test. **(B)** Enriched canonical pathways were identified for miR-142-3p targets using IPA software.

**FIGURE 7:**
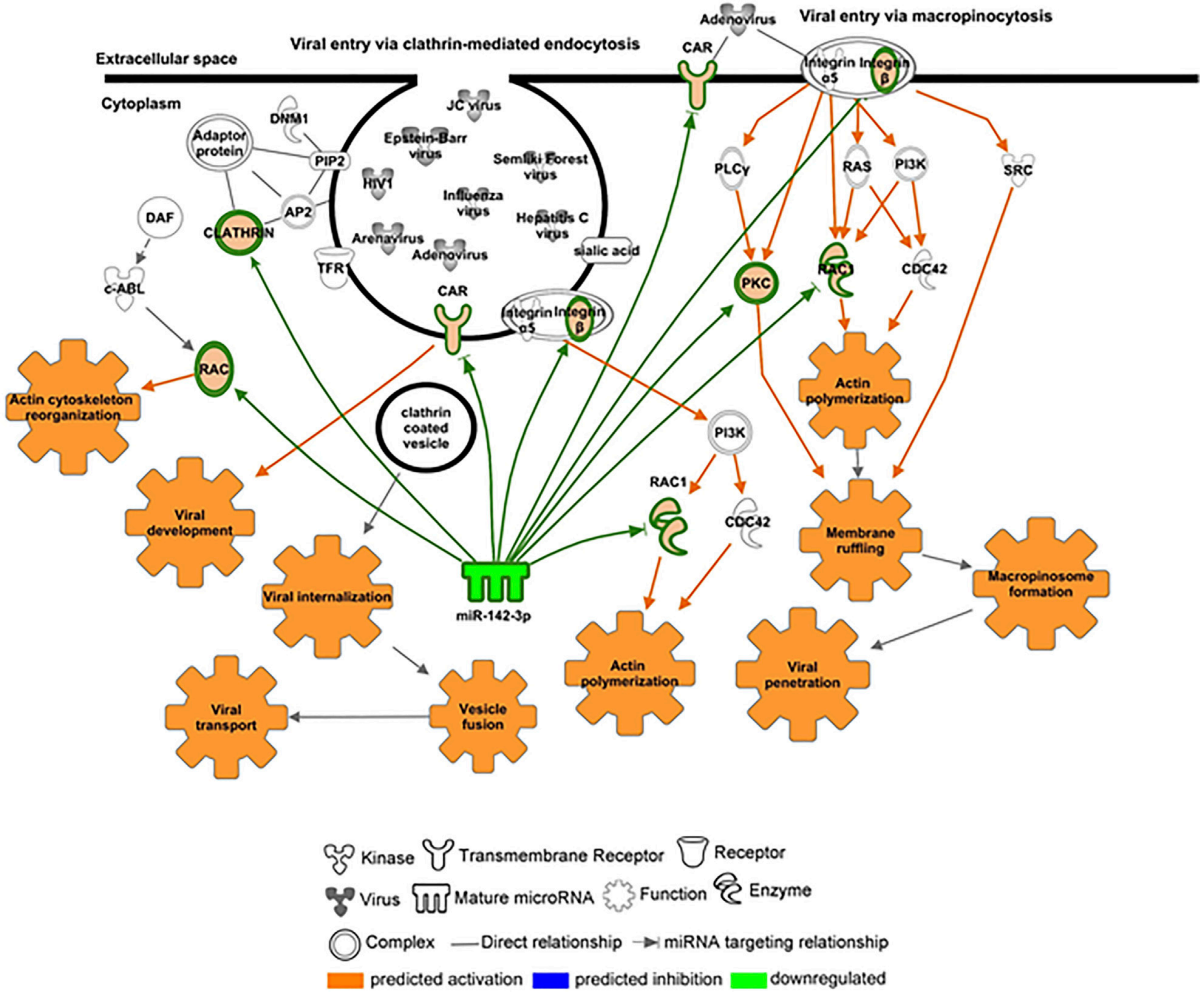
**–** Targets potentially regulated by miR-142-3p within the virus entry via an endocytic canonical pathway. Molecules highly predicted and experimentally validated as targets of miR-142-3p within the *virus entry via endocytic pathway* are shown as nodes with each node shape representing one type of molecule as indicated in the figure: clathrin, Rac family small GTPase 1 (RAC1), integrin beta 1, CXADR Ig-like cell adhesion molecule (CAR) and protein kinase C (PKC). Molecules and functions are colored based on their predicted activation status: activated (orange) or inhibited (blue) or decreased (gradient of green) or increased (gradient of red).

We further investigated the levels of miR-142-3p, specifically expressed in hematopoietic cells and with decreased levels in the plasma of ZIKV + patients. We first performed qPCR validation of miR-142-3p in a greater number of plasma samples, as shown in Figure 6A confirming its significant decreased levels in ZIKV + infected patients compared to CONTROL and RECZIKV + groups. Figure 6B shows the enriched canonical pathways for miR-142-3p targets. Among them, pathways related to endocytosis mechanisms: *clathrin-mediated endocytosis signaling,* and *virus entry via endocytic pathways* may indicate that miR-142-3p can potentially regulate intracellular trafficking and ZIKV + entry pathways. Figure 7 shows experimentally validated targets of miR-142-3p within the *virus entry via endocytic pathway*: clathrin, Rac family small GTPase 1 (RAC1), integrin beta 1, *CXADR* Ig-like cell adhesion molecule (CAR) and Protein kinase C (PKC). The prediction analysis showed that miR-142-3p could be potentially decreased by ZIKV infection and may interfere with the endocytic network activating process related to the virus intracellular trafficking incoming mechanism (in orange).

## Discussion

miRNAs are crucial post-transcriptional regulators which promote target messenger RNA decay or translational inhibition. In the past years, different studies have shown their biological importance in health and disease. It is also known that these small RNAs can be actively or passively present in different biofluids, including serum and plasma due to cell secretion or upon tissue damage, respectively. They can act as hormones mediating tissue crosstalk during physiological and pathogenic conditions and the study of circulating miRNA profiles can potentially reveal new biological mechanisms and disease biomarkers. Although miRNAs have been studied during Zika *in vitro* and *in vivo* infection, to the best of our knowledge, there is no study providing circulating miRNA profiling during Zika infection. In this study, we compared plasma miRNA profiles from Zika-infected patients during the acute (ZIKV+) and recovery phases of infection (RECZIKV+) compared to control individuals (CONTROL). Our results demonstrated that Zika-infected patients have a differential plasma miRNA profile compared to control individuals, characterized as a great number of DMs, most of them with decreased levels. Some recent studies have also shown a global miRNA downregulation in ZIKV-infected neurons *in vitro*, with few upregulated (Azouz et al., 2019). Here, we found that ZIKV + patients showed a decreased level of 22 and only two (miR-340 and miR-365-3p) with increased levels in their plasma. Dysregulation in miRNA expression has been previously shown to play critical roles during viral infections, controlling virus replication and modulating antiviral immunity (Louten et al., 2015; Xiong et al., 2017). ZIKV *in vitro* infection of human astrocytes was able to downregulate a great number of miRNAs, including the miR-30 family and miR-17-5p leading to deregulation of biological processes related to *unfolded protein response pathway* and interferon (IFNβ) production (Kozak et al., 2017). MiR-340-5p was previously described to be downregulated during *in vitro* infection with influenza A virus and mediates a regulatory feedback loop during host-virus interactions to control both antiviral responses and infection (Zhao et al., 2019). MiR-365-3p can negatively regulate interleukin 6 (*IL6*) gene expression (Xu et al., 2011), a central cytokine during acute phase response and associated with central nervous system protection during viral infections (Pavelko et al., 2003). MiR-146 is decreased when comparing ZIKV+ and RECZIKV + vs CONTROL group has an important role in *IFN signaling*. Wangs et al. have shown that miR-146 was able to suppress *STAT1*-dependent expression of type 1 and 2 interferons during HBV proliferation (Wang et al., 2013). Another important miRNA, miR-199 is decreased in plasma of the ZIKV + group, described as critical during HCV replication as its upregulation is related to increased viral replication (Fornari et al., 2010; Henry et al., 2010). Here, our clustering analysis revealed that when the patients pass from the acute to convalescent phase, their plasma miRNA content is similar to the CONTROL group, despite still having dysregulated miRNAs in common with the ZIKV + group. Computational analysis revealed that *senescence signaling* is a potential canonical pathway modulated by the dysregulated miRNAs observed in ZIKV + patients. Cellular senescence is described as a signaling pathway with dual opposite roles during cellular stress like viral infections. It can induce a proinflammatory phenotype and cell host protection (Baz-Martínez et al., 2016) but also can be used by the pathogen, like viruses, as a strategy to escape from the cellular antiviral system. The built network with targets of DMs from the ZIKV + group showed as a central node the embryonic lethal abnormal vision (ELAVL1), an RNA-binding protein (RBP) that is responsible for increasing the half-life and steady-state levels of different types of mRNAs, including the ones related to apoptosis and rapid inflammatory and stress response. *ELAVL1* mRNA (also named *HUR* or *ELAV1*) was previously shown to be an experimentally validated target of miR-125b-5p (Guo et al., 2009; Shwetha et al., 2018), also with decreased levels in the plasma of ZIKV + patients. The importance of this RNA-binding protein and miR-125 was previously highlighted during *in vitro* HCV infection. MiR-125 downregulation using antagomiRs led to an increase in the ELAV1 protein abundance in the cytoplasm enhancing HCV replication. This may be related to another described function of ELAV1 as an IFNΒ1 abundance regulator. ELAV1 strongly interacts with IFNΒ1 that, like most of the cytokines, contains adenylate-uridylate (A/U)-rich elements (ARES) which makes these cytokine mRNAs highly unstable. Based on that, reduced expression of *ELAVL1* downregulated type 1 *IFN* secretion and the first response to viruses (Herdy et al., 2015). In our study, miR-125 was not found to be dysregulated in the RECZIKV + group, and interferon signaling is among the enriched canonical pathways for this group. The top 2 most enriched pathways for RECZIKV+ were *oncostatin M signaling and the role of JAK family kinases* in IL6*-type cytokine signaling*. Both proteins are members of the same family sharing related receptor complexes and mediating communication between the central nervous system and the immune system. Among the targets identified for miR-30a-5p, which is decreased levels in both groups (ZIKV+ and RECZIKV+) compared to CONTROL, JAK1 was found within the *oncostatin signaling pathway*. In response to viral infections, the *JAK/STAT signaling pathway* is essential in the regulation of local inflammation (Ezeonwumelu et al., 2021). Oncostatin was described to play important roles during physiological and pathological conditions by maintaining neural precursor cell homeostasis and having neuroprotective action, respectively (Martins et al., 2019). Among the canonical pathways enriched in the list of shared DM, targets are the ones related to immune response and inflammation. For example, *neuroinflammation*, *toll-like receptor*, *the role of PRRs in recognition of viruses,* and IL-6 *signaling*. Finally, we highlighted the importance and previously described functions of miR-142-3p in the context of viral infections. This microRNA was first described by Chen et al., in 2004 as specifically expressed in embryonic and adult hematopoietic tissues, and is required for hematopoietic lineage development and function (Chen et al., 2004). MiR-142-3p was also shown to target cytokines like IL6 and ITGAV. Importantly, this miRNA interferes in viral replication, as previously shown in ZIKV-infected human umbilical cord mesenchymal stem cell assays (Seong et al., 2020). In addition, it may confer an antiviral defense as reported by Berrien-Elliott et al., 2019 for maintaining homeostasis and function of type I innate lymphoid cells (Berrien-Elliott et al., 2019). Enrichment analysis showed potential canonical pathways regulated by miR-142-3p. Among them, pathways related to endocytic mechanisms such as *clathrin-mediated endocytosis signaling and virus entry via endocytic pathways* indicate that this microRNA can potentially regulate intracellular trafficking and virus entry pathways. Among the highly predicted and experimentally validated targets of miR-142-3p are clathrin, Rac family small GTPase 1 (RAC1), integrin beta 1, and CXADR Ig-like cell adhesion molecule (CAR) and protein kinase C (PKC). The prediction analysis showed that the downregulation of miR-142-3p could be potently induced by a viral infection and will interfere with the endocytic network activating process related to the virus intracellular trafficking incoming mechanism. Our results showed a major decrease in miRNA levels in plasma infected with ZIKV. Importantly we further validated miR-142-3p decreased levels in a greater number of plasma samples. By identifying the targets of the DMs we listed important pathways potentially modulated by the dysregulated miRNAs. Furthermore, by building DM-target networks we could identify specific central molecules for the acute and recovery phases of ZIKV infection. A general limitation of the present study, given the network and pathway inference approaches, is the requirement of functional experimental validation to reliably infer interactions among nodes of the system. This is a particular obstacle in clinical studies where sample numbers and the ability to perform perturbations are often limited. Here, we present a tangential approach to reconstructing networks combining computational predictions, experimental evidence from large databanks, and literature mining integration. We think that the further study of miRNAs and their target molecules in the context of ZIKV infection may translate into the identification of novel therapeutic targets and biomarkers of recovery.

## Data Availability

The original contributions presented in the study are included in the article/Supplementary Material, further inquiries can be directed to the corresponding author.

## Supplementary Material

Supplementary Table S1 Clinical characteristics of the study subjects.

Supplementary Table S2 List of DMs in ZIKV+ vs CONTROL.

Supplementary Table S3 List of DMs in RECZIKV+ vs CONTROL.

Supplementary Table S4 List of DMs in common between ZIKV+ and RECZIKV+ .
