# Supplementary material for: A Major Downregulation of Circulating microRNAs in Zika Acutely Infected Patients: Potential Implications in Innate and Adaptive Immune Response Signaling Pathways
Source: Front Genet. 2022 Jun 1;13:857728. doi: 10.3389/fgene.2022.857728 (PMC9199004; doi:10.3389/fgene.2022.857728)
Supplement: Supplementary file 4 [file DataSheet1.pdf]

SUPPLEMENTARY MATERIALS

Supplementary Table 1 - Clinical characteristics of the study subjects

**Patient Code**	**Gender**	**Age** (years)	**Group**
C1	F	34	CONTROL
C2	F	36	CONTROL
C3	F	39	CONTROL
C4	F	36	CONTROL
C5	F	53	CONTROL
C6	F	37	CONTROL
C7	M	29	CONTROL
C8	M	27	CONTROL
C9	F	29	CONTROL
C10	M	58	CONTROL
C11[&]	M	34	CONTROL
C12	F	32	CONTROL
C13	M	27	CONTROL
C14	M	31	CONTROL
C15	M	39	CONTROL
C16	M	35	CONTROL
H17[&]	F	30	CONTROL
H18[&]	M	30	CONTROL
H19[&]	M	42	CONTROL
H20[&]	F	44	CONTROL
H21[&]	M	56	CONTROL
H22[&]	M	58	CONTROL
H23[&]	M	31	CONTROL
H24[&]	M	26	CONTROL
H25[&]	M	42	CONTROL
RECZIKV+1[#,&]	F	39	RECZIK+
RECZIKV+2[#,&]	F	36	RECZIK+
ZIKV+1/RECZIKV+3[,&]	F	36	RECZIK+/ZIKV+
ZIKV+2/RECZIKV+4[,&]	F	34	RECZIK+/ZIKV+
ZIKV+3/RECZIKV+5[,&]	F	49	RECZIK+/ZIKV+
ZIKV+4[#]	F	36	ZIKV+
ZIKV+5[#]	M	33	ZIKV+
ZIKV+6[#, &]	M	28	ZIKV+
ZIKV+7[#]	M	24	ZIKV+
ZIKV+8[&]	F	53	ZIKV+
ZIKV+9[&]	F	23	ZIKV+
ZIKV+10[&]	F	47	ZIKV+
ZIKV+11[&]	F	25	ZIKV+
ZIKV+12[&]	F	40	ZIKV+
ZIKV+13[&]	M	61	ZIKV+
ZIKV+14[&]	M	22	ZIKV+
ZIKV+15[&]	F	56	ZIKV+
ZIKV+16[&]	F	28	ZIKV+
ZIKV+17[&]	F	41	ZIKV+
ZIKV+18[&]	M	18	ZIKV+
ZIKV+19[&]	M	10	ZIKV+
ZIKV+20[&]	F	37	ZIKV+
ZIKV+21[&]	F	62	ZIKV+
ZIKV+22[&]	F	30	ZIKV+
ZIKV+23[&]	F	34	ZIKV+
ZIKV+24[&]	F	38	ZIKV+
ZIKV+25[&]	F	37	ZIKV+

Legend: Gender: F = female; M = male; CONT = individuals in the control group; ZIKAV+ = patients infected with Zika virus; REC-ZIKV+ = Zika-infected patients in convalescence phase. [#] Samples used for miRNA profilling. [&] samples used for miR-142-3p qPCR.

Supplementary Table 2 - List of DMs in ZIKV+ vs CONTROL

MicroRNA symbol/seed sequence	Mirbase ID	p value	fold change	log2 fold change
miR-365-3p (and other miRNAs w/seed AAUGCCC)	MIMAT0000710	0.034	5,77	2,53
miR-340-5p (miRNAs w/seed UAUAAAG)	MIMAT0004692	0.007	3,45	1,79
miR-342-3p (miRNAs w/seed CUCACAC)	MIMAT0000753	0.032	-2,69	-1,43
miR-30c-5p (and other miRNAs w/seed GUAAACA)	MIMAT0000244	0.002	-2,79	-1,48
let-7a-5p (and other miRNAs w/seed GAGGUAG)	MIMAT0000414	0.011	-2,90	-1,54
let-7e-5p (and other miRNAs w/seed GAGGUAG)	MIMAT0000066	0.015	-2,99	-1,58
miR-146b-5p (and other miRNAs w/seed GAGAACU)	MIMAT0002809	0.005	-3,22	-1,68
miR-126a-3p (and other miRNAs w/seed CGUACCG)	MIMAT0000445	0.007	-3,25	-1,70
miR-139-5p (miRNAs w/seed CUACAGU)	MIMAT0000656	0.007	-3,28	-1,71
miR-19b-3p (and other miRNAs w/seed GUGCAAA)	MIMAT0000074	0.034	-3,31	-1,73
miR-24-3p (and other miRNAs w/seed GGCUCAG)	MIMAT0000080	0.023	-3,40	-1,77
miR-320b (and other miRNAs w/seed AAAGCUG)	MIMAT0000510	0.014	-3,55	-1,83
miR-146a-5p (and other miRNAs w/seed GAGAACU)	MIMAT0000449	0.028	-3,69	-1,88
miR-186-5p (miRNAs w/seed AAAGAAU)	MIMAT0000456	0.006	-3,79	-1,92
miR-142-3p (and other miRNAs w/seed GUAGUGU)	MIMAT0000434	0.032	-3,94	-1,98
miR-30b (and other miRNAs w/seed GUAAACA)	MIMAT0000420	0.001	-3,95	-1,98
miR-199a-3p (and other miRNAs w/seed CAGUAGU)	MIMAT0000232	0.024	-4,31	-2,11
miR-125b-5p (and other miRNAs w/seed CCCUGAG)	MIMAT0000443	0.002	-4,41	-2,14
miR-191-5p (and other miRNAs w/seed AACGGAA)	MIMAT0000440	0.006	-4,63	-2,21
miR-223-3p (miRNAs w/seed GUCAGUU)	MIMAT0000280	0.035	-4,79	-2,26
miR-344a-5p (and other miRNAs w/seed CAGGCUC)	MIMAT0002174	0.007	-5,05	-2,34
miR-28-3p (and other miRNAs w/seed ACUAGAU)	MIMAT0004502	0.013	-5,13	-2,36
miR-150-5p (and other miRNAs w/seed CUCCCAA)	MIMAT0000451	0.019	-5,32	-2,41
miR-130a-3p (and other miRNAs w/seed AGUGCAA)	MIMAT0000425	0.014	-8,26	-3,05

Supplementary Table 3 - List of DMs in RECZIKV+ vs CONTROL

MicroRNA Symbol/seed sequence	Mirbase ID	p value	fold change	log2foldchange
miR-495-3p (and other miRNAs w/seed AACAAAC)	MIMAT0002817	0,01100	4,23	2,08
miR-17-5p (and other miRNAs w/seed AAAGUGC)	MIMAT0000103	0,01900	2,62	1,39
miR-146a-5p (and other miRNAs w/seed GAGAACU)	MIMAT0002809	0,04100	-1,53	-0,61
miR-342-3p (miRNAs w/seed CUCACAC)	MIMAT0000753	0,04400	-1,85	-0,89
miR-126a-3p (and other miRNAs w/seed CGUACCG)	MIMAT0000445	0,01700	-1,93	-0,95
miR-150-5p (and other miRNAs w/seed CUCCCAA)	MIMAT0000451	0,04600	-2,35	-1,23
miR-30c-5p (and other miRNAs w/seed GUAAACA)	MIMAT0000244	0,00400	-3,26	-1,70
miR-30b (and other miRNAs w/seed GUAAACA)	MIMAT0000420	0,00700	-3,29	-1,72

Supplementary Table 4 - List of DMs in common between ZIKV+ and RECZIKV+

MicroRNA Symbol/seed sequence	Mirbase ID	p value ZIKV+	p value RECZIKV+	fold change ZIKV+	fold change RECZIK+	log2foldchange ZIKV+	log2foldchange RECZIKV+
miR-126a-3p (and other miRNAs w/seed CGUACCG)	MIMAT0000445	0,007	0,017	-3,25	-1,931	-1,70	-0,95
miR-146a-5p (and other miRNAs w/seed GAGAACU)	MIMAT0000449	0,028	0,041	-3,69	-1,529	-1,88	-0,61
miR-150-5p (and other miRNAs w/seed CUCCCAA)	MIMAT0000451	0,019	0,046	-5,32	-2,347	-2,41	-1,23
miR-30c-5p (and other miRNAs w/seed GUAAACA)	MIMAT0000244	0,002	0,007	-2,79	-3,289	-1,48	-1,72
miR-342-3p (miRNAs w/seed CUCACAC)	MIMAT0000753	0,032	0,044	-2,69	-1,852	-1,43	-0,89
miR-30b (and other miRNAs w/seed GUAAACA)	MIMAT0000420	0,001	0,007	-3,95	-3,289	-1,98	-1,72
